# Complicated Hydatid Cyst Presentation: A Case Study on the Intersection of Cystobiliary Fistula, Bile Duct Obstruction, and Cholangitis: A Case Report

**DOI:** 10.1002/ccr3.71581

**Published:** 2025-12-01

**Authors:** Nasrin Razavianzadeh, Reza Dabiri, Aref Arminfar, Hessamedin Babaei, Faeze Gholipour, Farbod Noorbini, Soheil Shahramirad

**Affiliations:** ^1^ Department of Medical Sciences, Sha.C. Islamic Azad University Shahrood Iran; ^2^ Internal Medicine Department Semnan University of Medical Sciences Semnan Iran; ^3^ Student Research Committee, Shahrood Branch Islamic Azad University Shahrood Iran; ^4^ Department of Diagnostic Radiology Mashhad University of Medical Science Mashhad Iran

**Keywords:** CBD stone, cholangitis, Cystobiliary fistula, hydatid cyst

## Abstract

Early recognition of intrabiliary rupture in cystic echinococcosis is essential to prevent serious complications. Timely imaging and minimally invasive interventions such as endoscopic retrograde cholangiopancreatography, combined with antiparasitic therapy, can effectively manage biliary obstruction and reduce morbidity in complex cases.

## 1 Introduction

Cystic echinococcosis (CE), commonly referred to as hydatid disease, is a neglected zoonotic affliction attributed to infection by the larval stage of the cestode *Echinococcus granulosus* [1, 2]. This parasitic entity exhibits a broad geographical distribution, with endemic regions identified in western China, Central Asia, and South America, as well as Mediterranean and Middle Eastern countries [3]. Hydatid cysts primarily form in the liver but can also occur in other organs like the lungs, spleen, heart, and bone [4]. The clinical symptoms of hepatic hydatid cysts can vary significantly, ranging from mild to severe complications, including peritoneal rupture, cyst fistulization, and anaphylactic shock. The most frequently observed complication, present in approximately 50% of cases at the time of admission, is rupture of the cyst into the biliary tract, leading to secondary biliary obstruction caused by intracystic material or the development of cholangitis [5, 6]. In rare instances, rupture may lead to gastrointestinal involvement or cysto‐biliary communication (CBC). CBC remains an inadequately understood phenomenon, the exact etiology of which is not yet fully elucidated. However, it is postulated that this condition may arise due to inflammatory processes or increased pressure resulting from the enlargement of the cyst. Importantly, many cases are asymptomatic in the early stages of infection [7, 8, 9]. Due to the predominantly asymptomatic nature of many cases, hydatid cysts are frequently identified incidentally during radiological examinations [10]. The purpose of this case report is to elucidate a rare complication arising from an incidentally diagnosed hydatid cyst. This condition has the potential to manifest a diverse spectrum of serious complications, warranting further examination and understanding within the clinical context.

## 2 Case History/Examination

An 86‐year‐old male patient presented with jaundice, characterized by yellowing of the sclerae and skin, and persistent moderate epigastric pain that started 15 days ago. The pain disrupts his sleep intermittently but does not radiate. Exacerbations occur every two days and are accompanied by nausea, severe chills, and low‐grade fever. The patient has a history of constipation and has unintentionally lost about 3 kg in the past month. He denies shortness of breath, itching, or any skin issues, and reports no alcohol or tobacco use.

His medical history includes benign prostatic hyperplasia (BPH) and coronary artery bypass grafting, with no history of parasitic infections, recent travel, or pet ownership. He is currently taking medications for BPH and a heart condition. A review of other systems revealed no significant findings. The patient's vital signs were recorded as follows: a blood pressure of 120/80 mmHg, a respiratory rate of 17 breaths per minute without evidence of respiratory distress, a heart rate of 95 beats per minute, and a body temperature fluctuating between 38°C and 39°C. During the physical examination, tenderness was noted in the epigastric region and the right upper quadrant (RUQ) of the abdomen, although no abdominal distension was present. Murphy's sign was negative. Cardiac, pulmonary, and neurological examinations were conducted, revealing no abnormal findings.

## 3 Differential Diagnosis, Investigations, and Treatment

In the hematological parameters, a complete blood count (CBC) revealed leukocytosis, with a white blood cell count (WBC) of 17,000 cells/mm^3^. Electrolyte levels were within the normal range. The erythrocyte sedimentation rate (ESR) was measured at 110 mm/h. Additionally, the amylase level was found to be elevated at 336 U/L, while the lipase level remained within normal limits. Liver function tests indicated significant elevations, with aspartate aminotransferase (AST) at 428 U/L, alanine aminotransferase (ALT) at 501 U/L, alkaline phosphatase (ALK‐P) at 1288 U/L, gamma‐glutamyl transferase (GGT) at 253 U/L, total bilirubin at 5.1 mg/dL, and direct bilirubin at 2.8 mg/dL. Considering the patient's age and the potential for an underlying malignancy, the CA19‐9 tumor marker was evaluated, revealing levels exceeding 500 IU/mL.

A urinalysis was also conducted, which resulted in normal findings.

**FIGURE 1:**
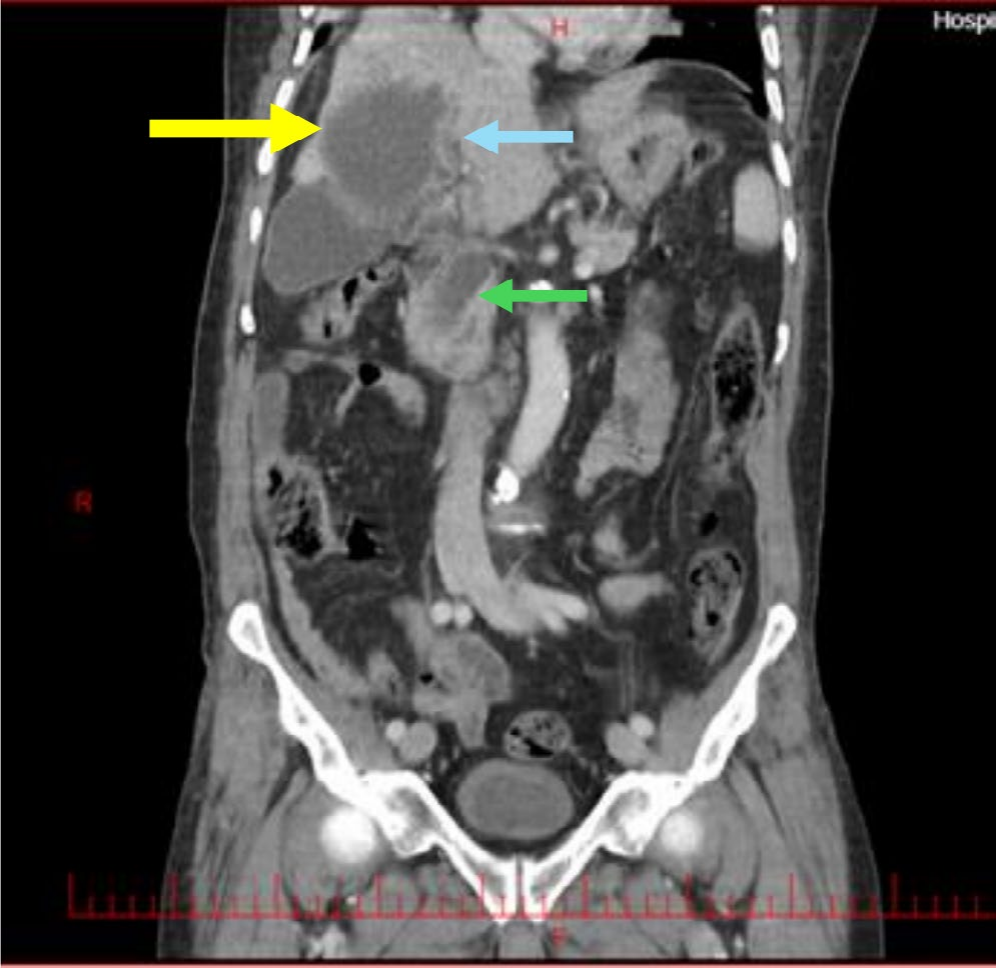
CT of the abdomen (coronal view). A hydatid cyst is observed in the right lobe of the liver (yellow arrow), alongside a fistula connecting the cyst to a branch of central biliary tree (blue arrow), which leads to the CBD (green arrow).

**FIGURE 2:**
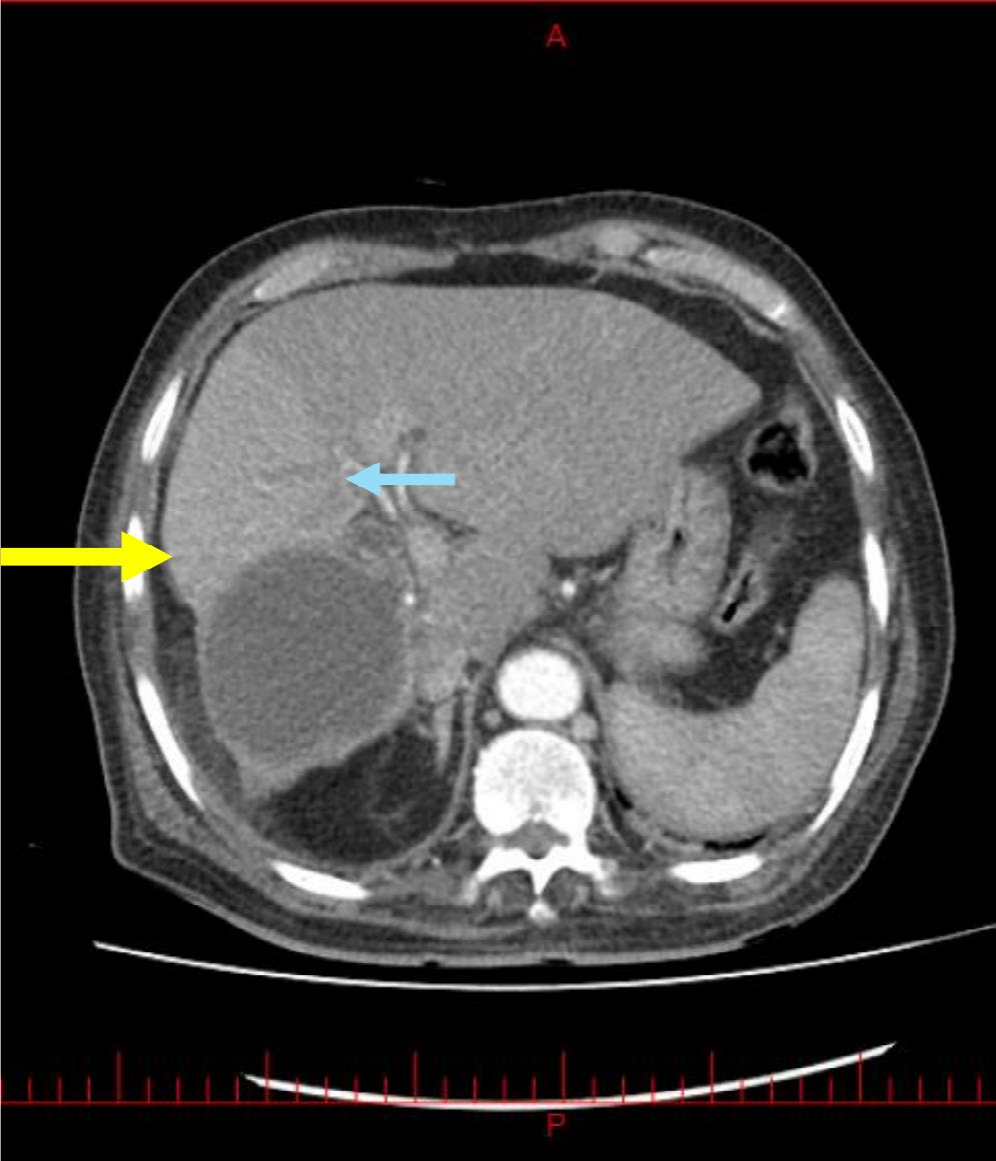
CT of the abdomen (axial view). A hydatid cyst is observed in the right lobe of the liver (yellow arrow), alongside a fistula connecting the cyst to a branch of central biliary tree (blue arrow), which leads to the CBD (green arrow).

In the initial ultrasound examination, a dilation of approximately 16 mm was observed in the common bile duct (CBD), with no stones detected. Additionally, an echogenic lesion was noted in the right hepatic lobe, prompting a recommendation for a triphasic computed tomography (CT) scan. Following this recommendation, a CT scan was performed, revealing a dilation of 20 mm in the CBD and the presence of a 10 mm stone located in the distal CBD (Figure 1). Furthermore, a cystic lesion measuring 70 × 63 mm was identified, characterized by thick‐walled, fluid‐filled lobulations with calcified walls, specifically located in segment VI of the right hepatic lobe (Figure 2). Upon identification of the stone and its location, along with the patient's clinical symptoms, a suspicion of acute cholangitis was raised, leading to a recommendation for endoscopic retrograde cholangiopancreatography (ERCP) for further evaluation and treatment.

Before the performance of ERCP, hypotension (systolic blood pressure of 90 and diastolic blood pressure of 60) and decreased level of consciousness were added to the previously established Charcot's triad. Consequently, to prevent further deterioration of the patient's condition, ERCP was promptly performed.

**FIGURE 3:**
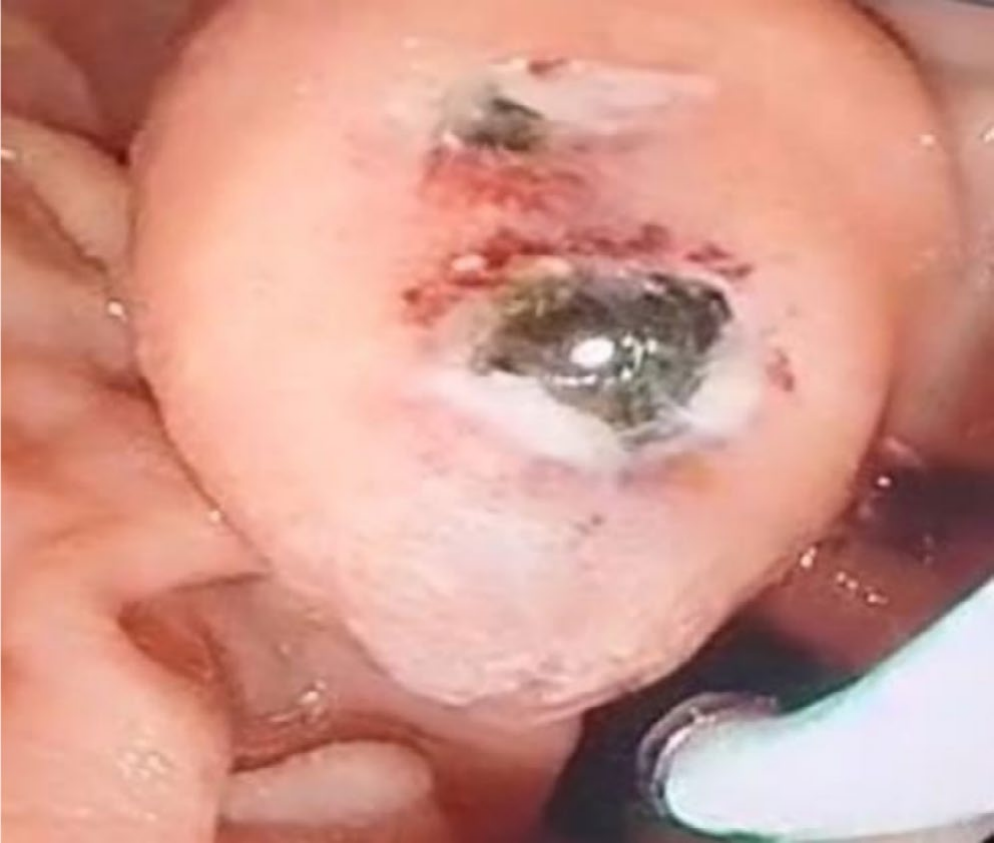
The major papilla, which is protruding into the duodenum, has the tip of a stone visible.

The procedure commenced with the identification of the major papilla, which was found to be protruding into the duodenum, with the tip of a stone visible (Figure 3).

**FIGURE 4:**
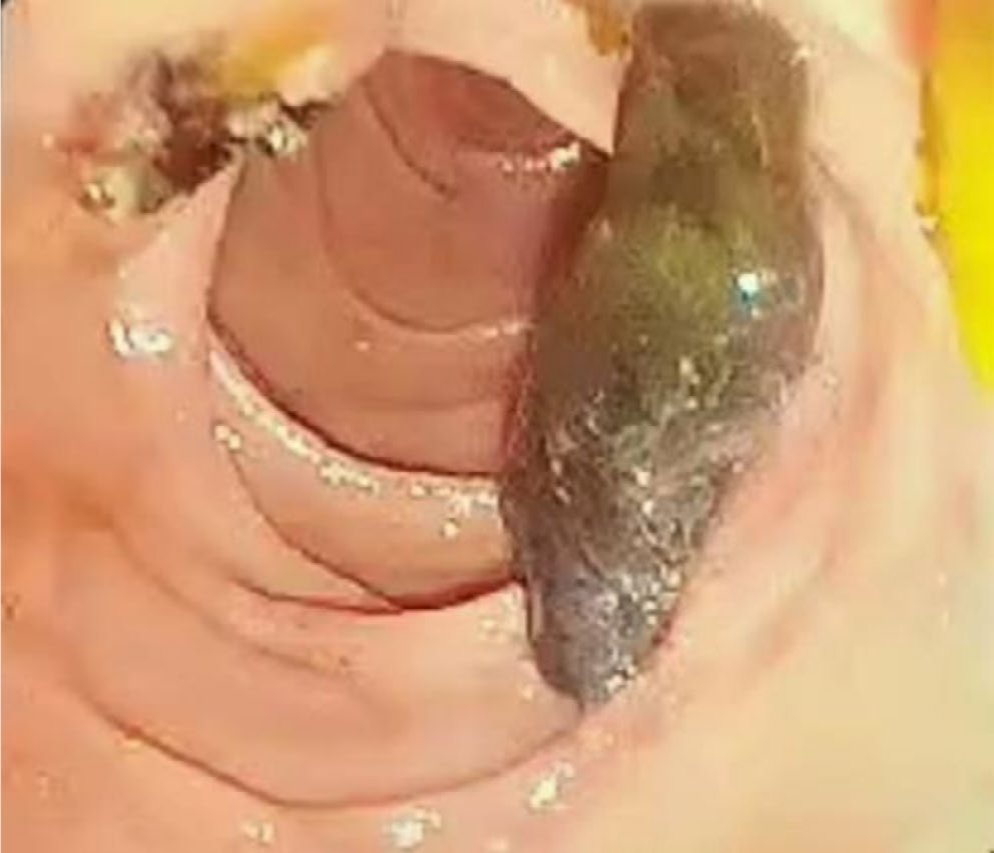
The release of a stone approximately 3 cm by 1 cm into the duodenum following sphincterotomy.

A precut sphincterotomy was performed, resulting in the spontaneous extraction of a large stone measuring approximately 3 cm by 1 cm (Figure 4). Following this, a second precut was executed, leading to the spontaneous removal of a sizable tubular cystic lesion, accompanied by a significant amount of thick bile and pus.

Subsequently, cannulation of the CBD was performed, and contrast material was injected, revealing a dilated CBD measuring 25 mm in diameter. Multiple balloon sweeps were conducted, during which some cystic particles were extracted. Finally, irrigation of the CBD with diluted epinephrine was carried out. The procedure was completed without any complications, including bleeding or perforation.

Following the inadvertent release of cystic debris into the patient's duodenum during ERCP (Figure 4), the clinical suspicion of a hydatid cyst was substantiated. An extensive evaluation of the patient's CT scan, along with the manifestation of purulent cholangitis and noticeable inflammation in the pancreaticobiliary duct, uncovered that this inflammatory process had led to the formation of a fistula between the duct and the hydatid cyst. This pathological development resulted in the duct penetrating the cyst, as illustrated in Figures 1 and 2. The identification of this complication highlights the critical need for accurate diagnosis and management in cases involving complex cystic lesions and associated inflammatory responses.

Due to the performance of ERCP and the subsequent observation of a fistula, the patient's CT scan was re‐evaluated (Figure 1). It was determined that the initial CT scan had indeed revealed the presence of cysto‐biliary communication; however, these findings had gone unrecognized by the radiologist. The ability to accurately diagnose such complications is critically dependent on the skill and precision of the radiologist, which plays an important role in the survival of the patient.

## 4 Outcome and Follow‐Up

Approximately one hour after the procedure, the patient's mental state and hypotension markedly improved. Subsequently, the patient was discharged following the completion of the postoperative follow‐up assessments. Liver function tests and bilirubin levels were normal, indicating a good prognosis. Oral medications were initiated on the fourth postoperative day and increased gradually. The patient continued albendazole for three months. The subsequent ultrasound examination revealed the presence of a cyst measuring 70 mm by 85 mm, characterized by a thickened wall and calcific foci. These imaging findings are suggestive of a type 3 hydatid cyst (dead phase).

## 5 Discussion

*Echinococcus species* are predominantly located in the liver (60%–80% of cases), followed by the lungs (20%–30% of cases), and other organs such as the spleen, brain, bones, heart (0.5%–2%), and kidneys (44%) [11, 12, 13]. These species exhibit varying incubation periods, which can extend for several years, contingent upon the cyst's anatomical location and the rate of its expansion [14]. Cystic echinococcosis, primarily caused by *E. granulosus* , is characterized by the presence of spherical, unilocular cysts that resemble a slowly enlarging tumor within the host organism. The cysts contain a clear fluid known as hydatid fluid [15, 16]. The clinical manifestations of cystic echinococcosis are highly dependent on the cyst's location within the body [16]. Patients may remain asymptomatic or exhibit a range of symptoms. In cases involving hepatic cysts, common symptoms include jaundice, fever, atypical abdominal pain and discomfort, and hepatomegaly accompanied by an abdominal mass [17]. Furthermore, the rupture of a cyst within the body—whether due to surgical intervention or trauma—can result in severe complications, including fever, pruritus, angioedema of the lips and eyelids, dyspnea, stridor, and potentially anaphylactic shock [11, 16, 18].

One type of rupture is the intrabiliary rupture (IBR), which occurs when a cyst opens into the biliary tract. This rupture may be presented as either occult or frank, with approximately 55%–60% of cases occurring within the biliary duct of the right lobe and 30%–35% in the biliary duct of the left lobe of the liver; it is rarely observed to open into the CBD. IBR can lead to a wide spectrum of clinical manifestations, depending on the size of the cyst, ranging from cholangitis, cholecystitis, and pancreatitis to septicemia. In our case, similar to the more common presentations, the ruptured cyst was also located in the right lobe of the liver [19, 20].

The rupture of a cyst into the biliary tree, along with the presence of tract stones, can lead to further dilation of the CBD and the manifestation of acute cholangitis symptoms, such as fever, abdominal pain, and jaundice. These symptoms were also observed in our case [21]. Given the variability in cyst sizes and the different types of fistulas, the diagnosis of CBC is often challenging, particularly when presurgical radiological examinations yield inconclusive results. This delayed diagnosis can lead to serious complications, including peritonitis and peritoneal abscesses [21, 22]. In the management of hydatid cysts, integrating radiologic findings with epidemiological context and relevant immunological data can substantially enhance diagnostic accuracy and support effective differentiation of cystic echinococcosis from other conditions [23].

Due to the observation of a stone in the CBD and the presence of cholangitis symptoms in the patient, our focus was directed toward performing ERCP to remove the stone. Although the exact cause of the CBC remains unclear, it is plausible to associate the etiology of this fistula with inflammation, particularly given the concurrent occurrence of cholangitis attributable to the stone in this case. Notably, these fistulas are typically identified either preoperatively or intraoperatively [6]. In a similar manner, our case exhibited penetration or leakage of a cyst into the CBD, which contributed to the need for ERCP, during which the cyst contents were discharged into the duodenum as a consequence of the CBC [24]. Initially, the presence of a fistula was not identified; however, after performing ERCP and re‐evaluating the CT scans, we discovered the fistula. Follow‐up CT scans revealed evidence of the fistula and the leakage of cyst contents into the ductal system (Figures 1, 2, 3). This emphasizes the importance of a thorough re‐evaluation of imaging studies, especially after procedures like ERCP, to identify complications that may not be immediately apparent.

Historically, the management of hydatid cysts and their associated complications relied heavily on surgical interventions, which were associated with increased morbidity and mortality rates. Recently, there has been a shift toward employing less invasive methods, such as ERCP, prior to surgical intervention to enhance the management of complications related to hydatid cysts [18]. In line with this, our case also utilized this less invasive approach for the drainage of cyst contents. When comparing surgical methods with less invasive approaches, recent evidence—including findings from our own case—indicates that both strategies are generally associated with good clinical outcomes and a low incidence of serious complications. This observation aligns with previous studies that have reported similarly favorable results across different treatment modalities. The benzimidazole pharmaceutical group, with albendazole being the most well‐known member, is utilized in the treatment of hydatid cysts. Medical treatment primarily aims to sterilize fistulas prior to surgical intervention and to prevent recurrence [25].

## Author Contributions

**Nasrin Razavianzadeh:** project administration. **Reza Dabiri:** investigation, project administration, writing – review and editing. **Aref Arminfar:** methodology. **Hessamedin Babaei:** investigation. **Faeze Gholipour:** data curation. **Farbod Noorbini:** conceptualization. **Soheil Shahramirad:** methodology, project administration, writing – review and editing.

## Funding

The authors have nothing to report.

## Ethics Statement

This case report was approved by the administration committee of ethics at the Faculty of Medical Sciences, Shahrood Islamic Azad University. The consent to participate in the study and for the publication of photographs was obtained from the patient's guardian.

## Consent

Written informed consent was obtained from the patient's legal guardian for publication of this case report and any accompanying images. A copy of the written consent is available for review by the Editor‐in‐Chief of this journal.

## Conflicts of Interest

The authors declare no conflicts of interest.

## Data Availability

The data that support the findings of this case report are available from the corresponding author upon reasonable request. All data are handled in accordance with the journal's data protection and privacy policies.
